# Preparation of Highly Dispersed Reduced Graphene Oxide Modified with Carboxymethyl Chitosan for Highly Sensitive Detection of Trace Cu(II) in Water

**DOI:** 10.3390/polym8040078

**Published:** 2016-04-01

**Authors:** Sheng Chen, Rui Ding, Xiuling Ma, Liqun Xue, Xiuzhu Lin, Xiaoping Fan, Zhimin Luo

**Affiliations:** 1 School of Ocean Science and Biochemistry Engineering, Fuqing Branch of Fujian Normal University, 1 Longjiang Road, Fuqing 350300, China; liqunxue@163.com (L.X.); linxiuzhu1030@163.com (X.L.); 2 College of Environmental Science and Engineering, Fujian Normal University, 8 Shangsan Road, Fuzhou 350007, China; rding@iue.ac.cn (R.D.); fanxiaopingfxp@163.com (X.F.); 3 College of Chemistry and Chemical Engineering, Fujian Normal University, 8 Shangsan Road, Fuzhou 350007, China; mxl502@163.com; 4 Jiangsu Key Laboratory for Organic Electronics & Information Displays and Institute of Advanced Materials (IAM), Nanjing University of Posts and Telecommunications, Nanjing 210046, China

**Keywords:** graphene, carboxymethyl chitosan, sensor, differential pulse anodic stripping voltammetry, Cu(II)

## Abstract

In this article, reduced graphene oxide (RGO)/carboxymethyl chitosan (CMC) composites (RGO/CMC) were synthesized by a hydrothermal method through *in-situ* reduction and modification of graphene oxide (GO) in the presence of CMC. An electrochemical sensor for the determination of Cu(II) by differential pulse anodic stripping voltammetry (DPASV) was constructed by an electrode modified with RGO/CMC. The fabricated electrochemical sensor shows a linear range of 0.02–1.2 μmol·L^−1^, a detection limit of 3.25 nmol·L^−1^ (S/N = 3) and a sensitivity of 130.75 μA·μmol·L^−1^·cm^−2^, indicating the sensor has an excellent detection performance for Cu(II).

## 1. Introduction

Copper is an essential element for human beings and plays an important role in various physiological processes at trace level [1,2]. However, due to its toxicity and non-degradation, the contamination of natural water by copper from mining, metal smelting and machinery manufacturing has become serious and attracted more attention [3,4]. The excessive copper intake from food chain can interact with lipid hydroxyperoxides, thereby disrupting cellular functions and causing extremely negative health diseases, such as Wilson’s disease and kidney damage [5,6,7,8]. Therefore, real-time, rapid and sensitive detection of Cu(II) in water environment is of significance [9]. Until now, there have been many detection methods for Cu(II), including flame atomic absorption spectrometry (AAS), ultraviolet-visible spectroscopy (UV-Vis), atomic fluorescence spectrometry (AFS) and inductively coupled plasma mass spectrometry (ICP-MS) [10]. Nevertheless, the tedious pretreatments like enrichment and extraction, and the high cost for these methods cannot meet the requirements of development of detecting heavy metal ion. Electrochemical analysis has become an ideal method for detecting metal ions in water due to the easy to transport apparatus, high sensitivity, fast response and low cost [11,12,13].

Graphene, the basic unit of carbonaceous materials, has been reported as an outstanding and promising material for the fabrication of electrochemical sensors due to its unique nanostructure, extraordinary electronic transport properties, excellent electrocatalytic activities and large surface area [14,15,16]. For instance, an electrochemical sensor fabricated by a gold electrode modified with graphene for the determination of Cu(II) and Pb(II) shows high sensitivity, good reusability and repeatability [17]. Wonsawat *et al.* [18] developed a bismuth-modified graphene-carbon paste electrode for detecting Cd(II) and Pb(II) in the automated flow system, and the detection limits reached 0.07 and 0.04 μg·L^−1^, respectively. However, the van der Waals and π-π stacking interactions between adjacent graphene sheets make them easy to agglomerate, which weakens the advantage of large surface area of graphene and limits its applications [19]. Chitosan, extracted from outer shells of shrimps, carbs and lobsters, was widely used for electrochemical determinations of metals ions [7]. Carboxymethyl chitosan (CMC) is a water-soluble and biodegradable derivate of chitosan, containing a large number of hydroxyl and carboxyl groups, which can make carbon nanomaterials such as carbon nanotube highly dispersed in the aqueous solution [20].

In this study, we prepared a functional nanocomposite through chemical modification of reduced graphene oxide with CMC. GO was reduced to RGO and *in-situ* modified with CMC in the procedure of synthesis. The chemical functionalization of RGO by CMC can efficiently inhibit the aggregation of RGO nanosheet in aqueous solution. RGO/CMC was used for modifying glassy carbon electrode (GCE) to detect Cu(II) in water by differential pulse anodic stripping voltammetry (DPASV). The results show that the electrochemical sensor has a linear range of 0.02–1.2 μmol·L^−1^, a low detection limit of 3.25 nmol·L^−1^ (S/N = 3), high sensitivity of 130.75 μA·μmol·L^−1^ cm^−2^, high selectivity and excellent reproducibility with the relative standard deviation (RSD) of 0.55%. Because RGO/CMC has high affinity towards metal ions and good conductivity, it has much potential for applications in the electrochemical sensors to detect trace Cu(II) in the water environment.

## 2. Materials and Methods

### 2.1. Chemical Reagents

Carboxymethyl chitosan (carboxylation degrees ≥60%) was purchased from Zhejiang golden shell biological chemical Co. Ltd. (Hangzhou, China). Graphite powder was purchased from Aladdin (Shanghai, China). Hydrazine hydrate (50%), potassium ferricyanide and glacial acetic acid (100%) were purchased from Guoyao Chemicals Co. Ltd. (Shanghai, China). Nafion (5%) was purchased from Sigma-Aldrich (St. Louis, MO, USA). 

### 2.2. Instruments and Measurements

The electrochemical experiments were performed on a CHI660D electrochemical workstation (CH Instrumental Co., Shanghai, China). The morphology of RGO/CMC was observed by transmission electron microscopy (TEM, JEM-2010F UHR, JEOL Ltd., Tokyo, Japan). The spectral properties were characterized by ultraviolet-visible spectroscopy (UV-Vis, TU-1800PC, Puxi Tongyong Instrument, Beijing, China), Fourier-transform infrared spectroscopy (FT-IR, Nicolet-380, Thermo Electron Co., Waltham, MA, USA), Raman spectra instrument (Olympus FV1000, Olympus Co., Tokyo, Japan). The surface property of RGO/CMC colloidal aqueous solution was studied by Zeta potential analyzer (Zetasizer Nano, Malvern Instruments Ltd., Worcestershire, UK). The component percentage of sample were measured through thermal gravimetric analysis (TGA, SDTA851e, Mettler-Toledo Co., Zurich, Switzerland) with a heating rate of 10 °C·min^−1^ using pure nitrogen as a carrier gas. The crystal structures of GO and RGO/CMC were characterized by X-ray diffraction (XRD, X’Pert, Philips Co., Eindhoven, The Netherlands).

### 2.3. Preparation of RGO/CMC Composite

GO was prepared by a modified Hummers method (See Supplementary Information). Two milliliters of GO (2 mg·mL^−1^), 2 mL of CMC (3 mg·mL^−1^) and 16 mL of deionized water were well mixed by ultrasonication for 2 h. Then, 1.4 µL of hydrazine hydrate were added to the solutions and the mixture was heated at 90 °C for 1.5 h. The reacted solution was filtered by microfiltration membrane (aperture < 0.22 µm) and washed by deionized water for at least three times. Finally, the as-prepared RGO/CMC sheet was dried at 60 °C for further use. 

### 2.4. RGO/CMC/Nafion Modified GCE

**Scheme 1:**
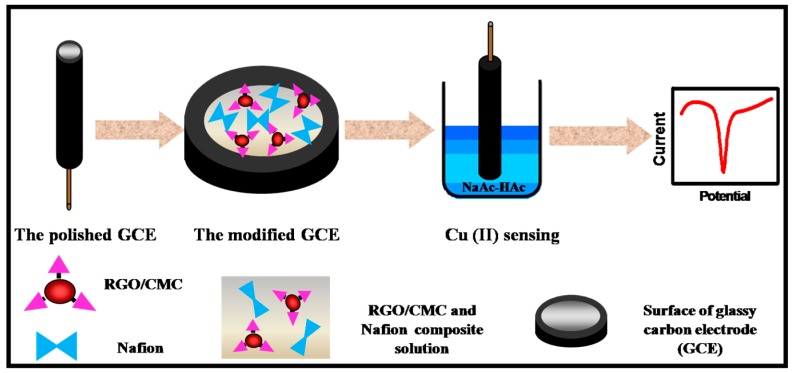
Schematic illustration of RGO/CMC/Nafion modified GCE for Cu(II) sensing.

As shown in Scheme 1, the fabrication of Cu(II) sensor was completed as briefly described in the following.

#### 2.4.1. Fabrication of Cu(II) Sensor by RGO/CMC/Nafion

Prior to the modification of electrode, the bare GCE was sequentially polished with 1.0, 0.3 and 0.05 µm alumina powder (α-Al_2_O_3_) and followed by ultrasonically rinsing with ethanol and deionized water in turn. 

The prepared RGO/CMC was dispersed in deionized water by ultrasonicaction. RGO/CMC/Nafion suspension was obtained by mixing the RGO/CMC solution (1 mg·mL^−1^) and Nafion solution (0.5%) with volume ratio of 1:1 through ultrasonication.

Then, RGO/CMC/Nafion was casted on the surface of GCE through dropping a certain amount of mixture suspension.

#### 2.4.2. Electrochemical Analysis

A standard three-electrode system connected to the CHI660E was used for Cu(II) detection. The RGO/CMC/Nafion modified GCE acted as the working electrode, an Ag/AgCl (saturated with KCl) as the reference electrode and a platinum wire as the counter electrode. The experiment was performed in a HAc-NaAc buffer solution (pH = 4.4) containing Cu(II). The electrochemical response of RGO/CMC/Nafion modified GCE was measured by DPASV with a scanning potential range from −0.50 to 0.40 V with a step voltage of 4 mV, a pulse amplitude of 50 mV, a pulse width of 0.06 s, and a pulse separation of 0.20 s.

The RGO/CMC/Nafion modified GCE was accumulated for 360 s by DPASV with stirring under the constant potential of −0.6 V. Afterwards, *i-t* method was used for restoring its activity by removing the sediment that adsorbed on the surface of the modified GCE at the potential of 0.60 V. 

## 3. Results and Discussion

### 3.1. Characterizations of RGO/CMC 

**Figure 1:**
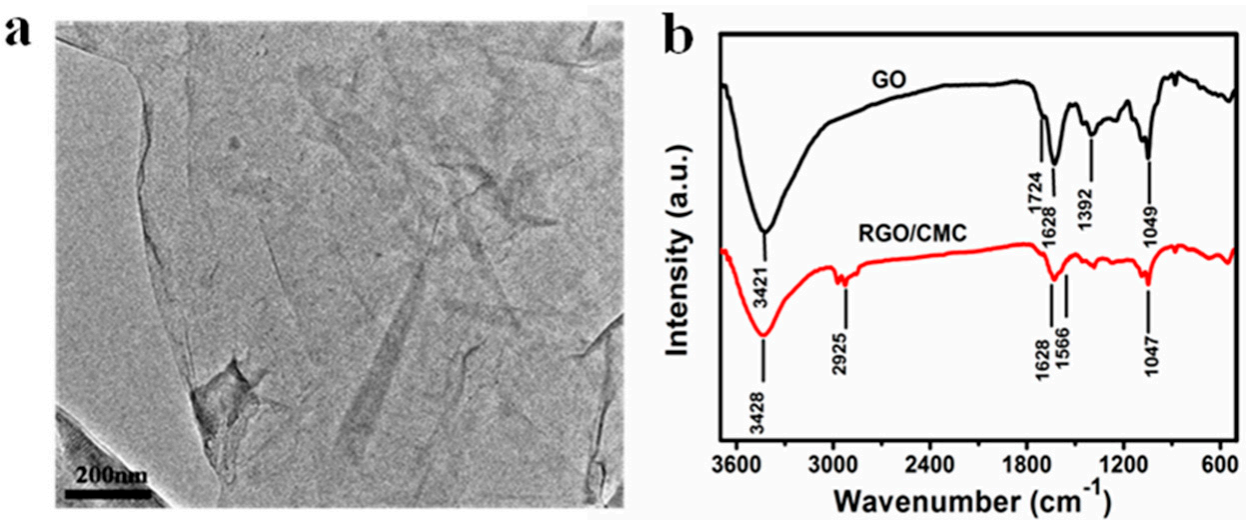
(**a**) A TEM image of RGO/CMC; (**b**) FT-IR spectra of GO and RGO/CMC.

As shown in Figure 1a, large RGO/CMC nanosheets are observed in the TEM image without agglomeration. FT-IR spectrum of RGO/CMC (Figure 1b) indicates that GO has been effectively reduced as the absorption intensity of the hydroxyl (3428 cm^−1^) and epoxy (1047 cm^−1^) were evidently decreased [21]. Furthermore, the new absorption peak appearing at 2925 cm^−1^ ascribed to the methyl and methylene of CMC, confirms the successful modification of CMC on the surface of RGO [22,23].

**Figure 2:**
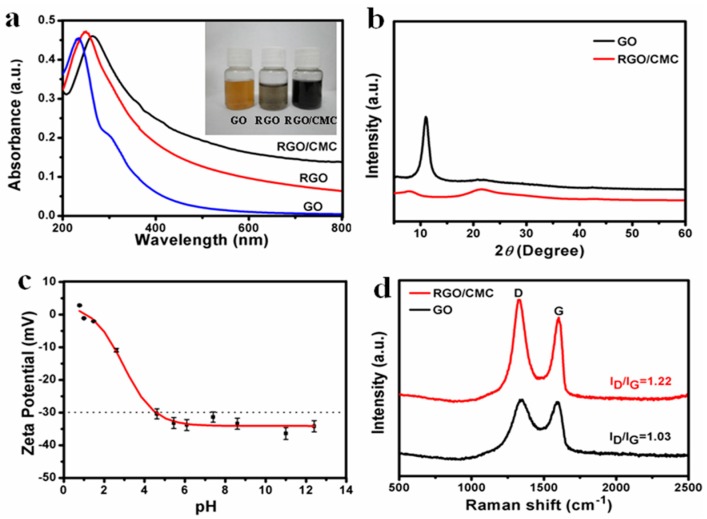
(**a**) UV absorption spectra of RGO/CMC, RGO and GO; (**b**) XRD diffraction patterns of GO and RGO/CMC; (**c**) Zeta potential of RGO/CMC aqueous solution; (**d**) Raman spectra of GO and RGO/CMC.

Figure 2a shows UV-Vis absorption spectra of GO, RGO and RGO/CMC. The spectrum of GO presents two characteristic features, a maximum peak at 242 nm assigned to π→π* transitions of aromatic C–C bonds and a shoulder peak around 301 nm assigned to n→π* transitions of C=O bonds [24]. After the reduction of GO, its maximum peak at 230 nm redshifts to 254 nm for RGO and 267 nm for RGO/CMC. Compared with GO, the obvious red-shift of maximum peak of the spectrum of RGO/CMC is due to the reduction of GO and thus restoration of electron [25]. The shoulder peak assigned to carbonyl bond absorption is no longer present because of its chemical reduction by hydrazine hydrate [26,27,28,29]. 

As seen in the XRD patterns (Figure 2b) of the GO and RGO/CMC, the pattern of GO exhibits a single peak at 11.05° corresponding to an interlayer *d* spacing of 8 Å, indicating the high degree of oxidation of GO [30]. Compared with GO, the XRD pattern of RGO/CMC shows a broad peak around at 22.35° (*d* = 4.16 Å). The breaking of the crystal structural integrity is the key factor for widening of the peak [31]. The restoration of electron may leads to the migration of peak [25]. Raman spectra of GO and RGO/CMC (Figure 2d) display two strong bands roughly at ~1346 cm^−1^ (D band) and at ~1590 cm^−1^ (G band) associated with the imperfection of the disordered sp^2^ bond structure of carbon materials and the mode of highly ordered pyrolytic graphite, respectively. Raman characterization indicates the main graphene structure of graphene is conserved in RGO/CMC composite [32]. The relative intensity ratio of D/G bands (*I*_D_*/I*_G_) of the GO and RGO/CMC are calculated to be 1.03 and 1.22, respectively, indicating a decrease in the average size of the sp^2^ domains after chemical reduction [33].

Zeta potential was used to evaluate the stability of colloidal solution. The higher the absolute value of Zeta potential is, the more stability the nanomaterials have. As shown in Figure 2c, the Zeta potential of RGO/CMC changes from 5 to −35 mV in the pH value range from 1.0 to 12.5. When the pH is between 4.5 and 12.5, the absolute value of Zeta potential is above 30 mV, illustrating the good stability of RGO/CMC in the pH value range from 4.5 to 12.5. According to the relevant literature [34,35], RGO can keep stable in alkaline environment (pH > 8), while CMC modified RGO can keep stable in a wider range of pH (pH > 4.5).

**Figure 3:**
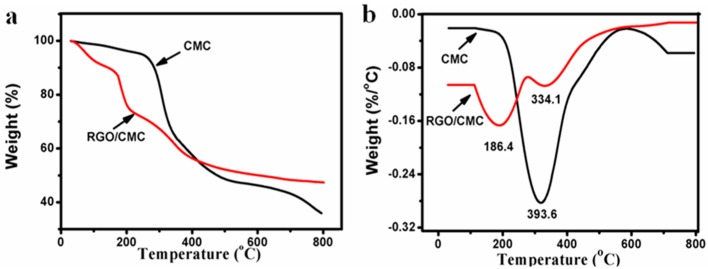
(**a**) TG and (**b**) DTG curves of CMC and RGO/CMC.

Figure 3 shows the thermal gravity analysis-differential thermal gravity (TG–DTG) curves of CMC and RGO/CMC. When the temperature rose from room temperature to 800 °C, a broad peak at 334.1 °C appeared in the DTG curve of CMC, corresponding to the 45% of mass loss step of TG curve assigned to the depolymerization and thermal decomposition of indican units in CMC at the temperature range of 250–400 °C [36]. In addition to the agravic peak at 334.1 °C, another obvious peak at 186.4 °C can also be observed from the DTG curve of RGO/CMC, indicating the decomposition of CMC in RGO/CMC [37]. When the temperature reaches to 500 °C, the TG curve of RGO/CMC changes slowly, and the residual mass loss of about 10% is mainly owing to the thermal reduction of graphene oxide. TG and DTG characterizations confirm that there is about 15 wt % of CMC on the surface of RGO.

### 3.2. Electrochemical Detection of Cu(II)

**Figure 4:**
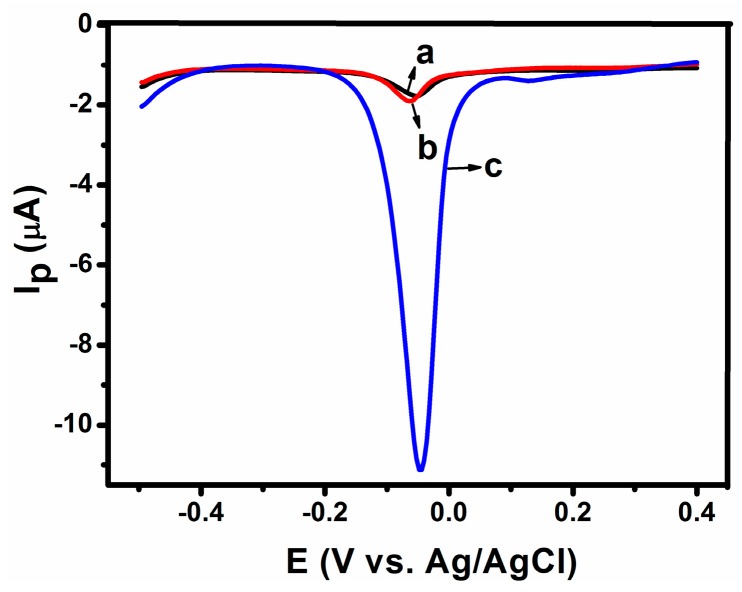
DPASV for 1.0 μmol·L^−1^ Cu (II) in 0.1 M NaAc-HAc (pH = 4.4) with different electrodes: (**a**) Bare GCE; (**b**) Nafion/GCE; and (**c**) RGO/CMC/Nafion/GCE. Detection conditions: amplitude of 0.05 V, pulse width of 0.06 s, and pulse period of 0.20 s.

For comparison, electrochemical experiments of bare GCE, GCE/Nafion and GCE modified with RGO/CMC/Nafion were carried out by DPASV under the same experimental conditions. Figure 4 depicts the detection performances of electrodes for 1.0 μmol·L^−1^ Cu(II) in 0.1 mol·L^−1^ NaAc-HAc (pH = 4.4). After accumulation for 360 s, the anodic peak current response of the RGO/CMC/Nafion modified GCE (c in Figure 4) is higher than bare GCE (a in Figure 4) and GCE/Nafion (b in Figure 4), indicating RGO/CMC can dramatically improve electroanalytical current response for Cu(II). According to the research of Khomyakov *et al.* [38], the interaction and charge transfer between graphene and metal ions is one of the main reasons for sensitivity of modified electrode. The detection of Cu(II) by the RGO/CMC/Nafion modified GCE may be by two steps and the reaction equations are shown below:

(1) The enrichment process.

(Cu(II)) sol. + (M) surf. — (Cu(II)-M) ads. (Cu(II)-M) ads. + 2e^−^ — (Cu(0)-M) ads.

(2) The dissolution process.

(Cu(0)-M) ads. — (Cu(II)) sol. + (M) surf. + 2e^−^

M in the equation stands for RGO/CMC/Nafion.

During the enrichment process, the chelated Cu(II) will be reduced to elementary copper at the working potential of 0.60 V. After enrichment, with the potential changes from −0.50 to 0.40 V, the copper dissolves and a sensitive anodic stripping peak can be seen at 0.06 V.

The peak potential of RGO/CMC/Nafion modified GCE has a slightly negative shift of −0.06 V (c in Figure 4) as compared to GCE (a in Figure 4) and Nafion/GCE (b in Figure 4), which may due to the accelerated electron transfer process by the functionalization of RGO/CMC [39]. The higher anodic peak current response can be attributed to the function of RGO/CMC, which can offer more sites to chelate Cu(II) for improving current response because of abundant carboxyl groups in CMC and RGO.

### 3.3. Optimization of Detection Conditions

**Figure 5:**
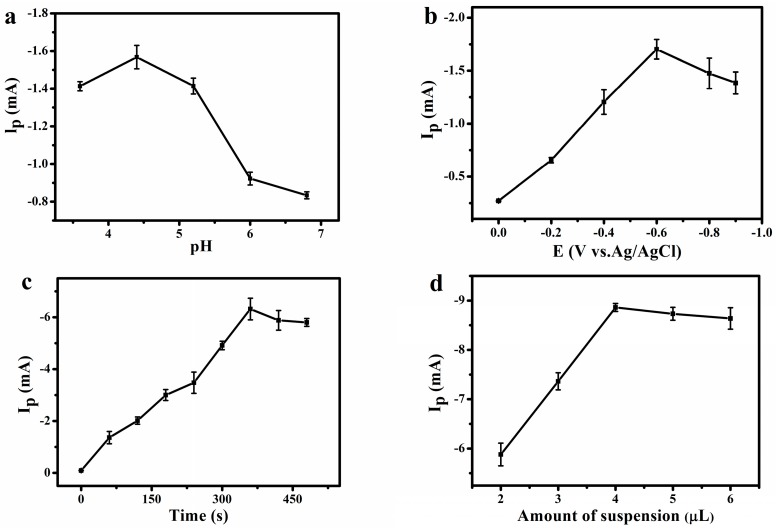
The effect of: (**a**) pH; (**b**) accumulation potential; (**c**) accumulation time; and (**d**) dosage of RGO/CMC/Nafion on the stripping peak current of 1.0 × 10^−6^ mol·L^−1^ Cu(II).

Table 1 shows the effect of various supporting electrolytes, including acid solution, alkaline solution and neutral solution, on different pulse anodic stripping peak current of the Cu(II) on RGO/CMC/Nafion/GCE. It is found that the stripping peak current in NaAc–HAc (0.1 mol·L^−1^) is the highest, followed by the hydrochloric acid solution, sulfuric acid, potassium chloride, and no response in the sodium hydroxide solution. The electrochemical responses of Cu(II) in different pH values of NaAc–HAc (0.1 mol·L^−1^) at RGO/CMC/Nafion/GCE were studied by DPASV. As show in Figure 5a, the stripping peak current of the modified GCE increases with the increase of pH value from 3.6 to 4.4. After that, the peak current decreases with the further increase of pH value, which is consistent with the results of Table 1. Consequently, considering the sensitivity and stability of detection, NaAc–HAc (0.1 mol·L^−1^) was chosen for this work. 

The influences of accumulation potential, accumulation time and dosage were also investigated. The effect of accumulation potential on peak current is illustrated in Figure 5b. The peak current increases rapidly with the decrease of accumulation potential from 0.0 to −0.60 V. It can be explained that Cu(II) is able to be reduced at more negative potential. However, with the further decrease of accumulation potential from −0.60 to −0.90 V, the peak current decreases gradually. Parts of the active sites on the modified electrode surface are occupied by some other ions in the negative potential, leading to obstruction of the determination of Cu(II). Figure 5c displays the influence of accumulation time for stripping peak currents. When the accumulation time is 360 s, the response current is the highest because the adsorption of Cu(II) on the surface of modified electrodes has reached saturation [40]. The addition of RGO/CMC/Nafion was studied in the range of 2–6 μL. As shown in Figure 5d, when the dosage of RGO/CMC/Nafion is 4 μL, the response current is the highest. When the dosage continue to increases, the current decrease, which is mainly due to the increase of film thickness obstructing the electron transfer process between Cu(II) and electrode. 

The repeated use of RGO/CMC/Nafion modified electrode was examined by *i-t* measurement. Table 2 shows the change of peak currents after repeated measurement of 1.0 × 10^−6^ mol·L^−1^ Cu(II) for using the same RGO/CMC/Nafion modified electrode ten times. The relative standard deviation is 0.55%, indicating the RGO/CMC/Nafion modified GCE has an excellent reproducibility for the detection of Cu(II).

### 3.4. Anti-Interference of RGO/CMC/Nafion Modified GCE

Since there are still some other common metal ions in water, the detection of the identification performance of RGO/CMC/Nafion modified GCE is necessary. For practical purposes, 1.0 × 10^−6^ mol·L^−1^ Cu(II) solutions with different metal ions commonly presenting in natural water were used to examine the anti-interference of RGO/CMC/Nafion modified electrode. As shown in Table 3, the response current of the RGO/CMC/Nafion modified GCE changes within less than ± 5% with the addition of the interfering ions. Therefore, the RGO/CMC modified GCE is suitable for the detection of Cu(II) in real water samples after some pretreatments.

### 3.5. Detection Limit of Cu(II) with RGO/CMC/Nafion Modified GCE

**Figure 6:**
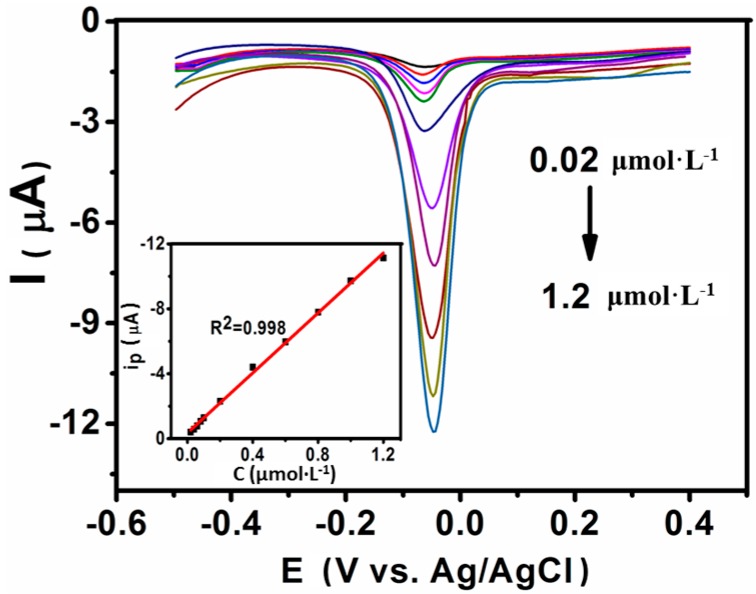
DPASV of the RGO/CMC/Nafion modified GCE in 0.1 M NaAc–HAc (pH = 4.4) with various concentrations of Cu(II). From top to bottom, the different colors of the curves represent the concentration of Cu(II) of 0.02, 0.04, 0.06, 0.08, 0.1, 0.2, 0.4, 0.6, 0.8, 1.0 and 1.2 μmol·L^−1^, respectively. Inset is the calibration curve of the stripping peak currents *versus* the concentrations of Cu(II).

Figure 6 shows the stripping voltammograms under the optimized conditions with the concentration of Cu(II) from 0.02 to 1.2 μmol·L^−1^ and the corresponding calibration curve of the stripping peak current *versus* the concentrations of Cu(II) (inset). The RGO/CMC/Nafion modified electrode shows good detection limit of 3.25 nmol·L^−1^ (S/N = 3) and a sensitivity of 130.75 μA·μmol L^−1^·cm^−2^. The limit of detection (LOD) was calculated as follows: LOD = 3 S/m, where S is standard deviation of current value, m is sensitivity, which is the slope of the linear equation. Comparison of other modified electrodes for the determination of Cu(II) is given in Table 4. The present work exhibited better electrochemical analysis performance in detecting trace Cu(II) with lower detection limit in wide linear range.

## 5. Conclusions

In this article, water-soluble and highly dispersed RGO/CMC was prepared by the chemical reduction of GO and *in-situ* modification with CMC. A simple and effective electrochemical sensor for determination of Cu(II) was constructed by DPASV based on the RGO/CMC/Nafion modified GCE. The RGO/CMC/Nafion modified GCE displays good analytical performance including wide linear range, low detection limit, high sensitivity, good repeatability to Cu(II) and excellent anti-interference ability towards other interfering metal ions. The fabricated sensor based on RGO/CMC/Nafion is promising in the determination of trace Cu(II) in real water samples.

## Figures and Tables

**Table 1:** Effect of different types of supporting electrolytes on the stripping peak currents.

Supporting Electrolyte	Stripping Peak Current (μA)
KCl (0.1 mol·L^−1^)	0.3670
NaOH (0.1 mol·L^−1^)	No response
H_2_SO_4_ (0.1 mol·L^−1^)	0.9271
HCl (0.1 mol·L^−1^)	1.056
NaAc–HAc buffer solution	1.568

**Table 2:** *i_p_* of the RGO/CMC/Nafion modified GCE responding to the 1.0 × 10^−6^ mol·L^−1^ of Cu(II).

N	1	2	3	4	5	6	7	8	9	10
*i*_p_ (μA)	0.723	0.711	0.718	0.714	0.719	0.719	0.722	0.716	0.721	0.723

**Table 3:** (*i*_o_ − *i*_p_)/*i*_p_ of sensor for 1.0 × 10^−6^ mol·L^−1^ Cu(II) in the presence of other metal ions.

Concentration (mol·L^−1^)	Species	Interference (%)
5.0 × 10^−4^	Na^+^	−3.5
	K^+^	−2.9
	Ca^2+^	−4.8
	Mg^2+^	−1.5
1.0 × 10^−4^	Mn^2+^	−3.1
	Cd^2+^	−4.6
2.0 × 10^−5^	Pb^2+^	−4.9
	Zn^2+^	−4.4

**Table 4:** Comparison of different modified electrodes for detecting Cu(II).

Modifier	Electrode	Method	Detection Range (μmol·L^−1^)	Detection Limit (nmol·L^−1^)	Ref.
Tripeptide (Gly-Gly-His)	GCE	DPSV	0.1–30	46	[41]
AMT-g-NGO	CPE	SWASV	0.1–1.0 × 10^5^	40	[42]
Graphene	Gold electrode	OSWV	1.5 × 10^−3^–0.02	1.5 ± 0.2	[17]
Propargyl-functionalized ferrocene	Gold electrode	DPV	1.0 × 10^−8^–1.0 × 10^−3^	3.4 × 10^−6^	[43]
Ionic liquid-functionalized orderd mesoporous silica SBA-15	CPE	DPASV	0.3–100	10	[44]
Crosslinked chitosan	CNPE	LSASV	0.079–16	10	[7]
Silica	CPE	DPSV	0.05–0.2	3	[45]
RGO/CMC	GCE	DPASV	0.02–1.2	3.25	Present work

DPSV: Differential pulse stripping voltammetry; AMT-g-NGO: 2-amino-5-mercapto-1,3,4-thiadiazole/Nano graphene oxide; CPE: Carbon paste electrode; SWASV: Square wave anodic stripping voltammetry; OSWV: Osteryoung square wave voltammetry; DPV: Different pulse voltammetry; CNPE: Carbon nanotubes past electrode; LSASV: Linear scan anodic stripping voltammetry.

## Supplementary Materials

Supplementary materials can be found at www.mdpi.com/2073-4360/8/3/78/s1.
