# Supplementary material for: Preparation of Highly Dispersed Reduced Graphene Oxide Modified with Carboxymethyl Chitosan for Highly Sensitive Detection of Trace Cu(II) in Water
Source: Polymers (Basel). 2016 Apr 1;8(4):78. doi: 10.3390/polym8040078 (PMC6432173; doi:10.3390/polym8040078)
Supplement: Supplementary file 1 [file polymers-08-00078-s001.pdf]

SupplementaryMaterials: Preparation of Highly Dispersed Reduced Graphene Oxide Modified with Carboxymethyl Chitosan for Highly Sensitive Detection of Trace Cu(II) in Water

Sheng Chen, Rui Ding,Xiuling Ma, Liqun Xue, Xiuzhu Lin, Xiaoping Fan and Zhimin Luo

The preparation of graphene oxide (GO): Graphite oxide was prepared from natural graphite powder by a modification of Hummers. In brief, 1.0 g of graphite powder and 30 mL of sulfuric acid were added into a reaction vessel in a dry ice bath, and stirred gently for 6 h. Then, 3.0 g of potassium permanganate was added slowly with vigorous stirring. The reaction was allowed to proceed at below 20 °C for 30 min and at 35 °C for 30 min. Then, 30 mL of deionized water was added into the reaction vessel slowly, and the reaction was kept at ~95 °C for 35 min. Finally, 140 mL of deionized water and 10 mL of 30% hydrogen peroxide were added into the reaction vessel to finish the reaction. The resulting graphite oxide was filtered and washed using 5% hydrochloric acid and deionized water to remove the free SO_4^{2-}. The graphite oxide was suspended in the deionized water, and exfoliated through ultrasonication for 3 h. The colloidal solution was centrifuged at the speed of 5000 rpm for 10 min to remove the unexfoliated graphite oxide. The yellow-brown upper solution was graphene oxide, which must be dialyzed and freeze dried.
